# Charged Functional Groups Drive Nanofiltration Li^+^/Mg^2+^ Selectivity

**DOI:** 10.3390/membranes16080264

**Published:** 2026-08-10

**Authors:** Suwei Liu, Jiaxuan Wang, Sinan Keten, Richard M. Lueptow

**Affiliations:** 1 Department of Mechanical Engineering, Northwestern University, Evanston, IL 60208, USA; suweiliu2017@u.northwestern.edu (S.L.); s-keten@northwestern.edu (S.K.); 2 Mercersburg Academy, Mercersburg, PA 17236, USA; wangj27@mercersburg.edu; 3 Department of Civil and Environmental Engineering, Northwestern University, Evanston, IL 60208, USA; 4 Department of Chemical and Biological Engineering, Northwestern University, Evanston, IL 60208, USA

**Keywords:** molecular dynamics, solute transport, solute rejection, water filtration membrane, selectivity

## Abstract

Polyamide nanofiltration (NF) membranes offer a scalable and energy-efficient pathway for lithium concentration from lake brines and battery leachates, but practical implementation hinges on achieving high selectivity of Li^+^ over Mg^2+^. The active layer of these membranes can be positively or negatively charged, carrying both amine groups that can be protonated and carboxyl groups that can be deprotonated with an ionization state that is set by the feed pH. Here, molecular dynamics simulations are used to elucidate how pH-dependent charged functional groups within the polymeric nanostructure of NF membranes govern Li^+^/Mg^2+^ selectivity, arising from electrostatic charge interactions between ions and functional groups at the molecular scale as well as steric size exclusion within the membrane pore structure. Although single-salt Li^+^ or Mg^2+^ feed solutions exhibit similar ion penetration behavior, mixed Li^+^/Mg^2+^ feeds show markedly enhanced Li^+^/Mg^2+^ selectivity at low concentrations when the membrane is positively charged. This selectivity arises because Mg^2+^ interacts more strongly than Li^+^ with repulsive protonated amine (NH_2_^+^) groups, suppressing divalent ion transport, an effect that emerges specifically when the two cations compete for the same ${\mathrm{Cl}}^{-}$ ions in a mixed feed. In contrast, for negatively charged membranes, attractive interactions with deprotonated carboxylate (${\mathrm{COO}}^{-}$) groups strongly hinder the transport of both ions, resulting in poor selectivity. Ion clustering within membrane pores further reduces transport through steric effects. These results provide molecular-level insight into how charged functional groups control mono/divalent ion selectivity when competing ions are present and highlight positively charged membranes as optimal platforms for lithium separation applications.

## 1. Introduction

The rapid growth of lithium-ion battery technologies has intensified demand for scalable and energy-efficient methods to concentrate and recover lithium from natural and industrial sources, including salt lake brines and spent battery leachates. Nanofiltration (NF) membranes are particularly attractive for these emerging applications because they enable continuous operation at low energy cost and can be readily integrated into existing separation processes [1,2,3,4,5,6,7,8,9,10]. However, practical implementation is fundamentally limited by the difficulty of selectively transporting Li^+^ over Mg^2+^, since magnesium ions are typically present and can severely reduce lithium recovery efficiency. Polyamide NF membranes are also crucial for addressing global water scarcity where, as with Li^+^ separation, their unique ability to selectively remove divalent ions and large molecules at low energy cost [11,12,13] is particularly important for brackish water desalination and wastewater treatment [14,15].

Although NF membranes rely in part on size exclusion mechanisms, membrane charge plays a fundamental role in determining ion selectivity in polyamide membranes via electrostatic and Donnan exclusion mechanisms [16,17], which operate at the angstrom scale in polymeric membrane materials. For lithium recovery, enhancing Li^+^/Mg^2+^ selectivity therefore requires precise control of membrane charge and its interaction with competing ions. However, ion interactions with charged polyamide membrane nanostructures are not well understood, particularly when multiple ions are present [18,19,20], even though this is essential for optimizing NF membranes in terms of membrane electrostatic charge level and heterogeneity [21,22]. In particular, feed water pH alters the ionization state of ${\mathrm{COO}}^{-}$ and NH_2_^+^ functional groups within the membrane’s polymeric nanostructure, leading to changes in the magnitude and spatial distribution of charged groups in the membrane [23,24]. This, in turn, alters ion transport and rejection in the membrane, which affects ion selectivity when multiple ion species are present, as is the case for lithium-ion recovery applications.

Conventional continuum-level experiments such as zeta potential measurements and ion rejection tests [25,26,27,28] provide valuable macroscopic insight but cannot resolve the sub-nanosecond and sub-angstrom scales of solute ion interactions with membrane charged groups [29,30,31,32]. Similarly, continuum transport theories [33,34,35] capture average electrostatic effects but neglect molecular-level phenomena such as ion-functional group pairing, transient clustering, and spatial heterogeneity of membrane charge. The consequence is an inadequate understanding of the molecular-level interactions of Li^+^ and Mg^2+^ ions with ${\mathrm{COO}}^{-}$ and NH_2_^+^ functional groups in charged NF membranes. As a result, the mechanistic origins of Li^+^/Mg^2+^ selectivity, as well as mono/divalent selectivity for other ions, remain poorly understood.

Molecular dynamics (MD) simulations provide a powerful complementary framework for probing ion–membrane interactions at the molecular-level in a virtual pH-dependent charge environment. MD simulations enable the exploration of molecular interactions between solutes and the membrane under controlled conditions with sub-angstrom and sub-nanosecond resolution. This atomistic approach allows quantitative analysis of the local membrane density and porosity, trajectories of individual solute ions, and interaction energies between ions and the membrane charged groups. Consequently, MD simulations can reveal mechanisms that are difficult to capture through continuum-level experiments or theory. Previous MD studies of reverse osmosis (RO) membranes of trimesoyl chloride (TMC) polymerized with m-phenylene diamine (MPD), as well as NF membranes of TMC polymerized with piperazine (PIP), have proven invaluable in unraveling the intricate mechanisms governing water and solute transport in membrane materials at the molecular scale [36,37,38,39,40].

In this paper, we use MD simulations to systematically investigate ion–membrane charge interactions with mixed feed solutions containing both LiCl and MgCl_2_. This study extends and expands our previous work on the much simpler problem of such interactions with single solute monovalent (LiCl) and divalent (MgCl_2_) feed solutions [8] to explore how ${\mathrm{COO}}^{-}$ and NH_2_^+^ charged groups within the polymeric membrane nanostructure interact with multiple ions to influence their transport. The two key advances over our previous single-salt studies are: (i) a molecular-level characterization spanning the full range of pH-induced charge states, including the strongly negative membrane at pH = 10 where cation transport is effectively arrested, a regime not previously characterized at this resolution; and (ii) the demonstration that competitive interactions between Li^+^ and Mg^2+^ in a mixed feed produce selectivity behavior that cannot be discerned from single-salt simulations. Because MD simulations allow individual variables to be isolated and controlled, we focus specifically on ion selectivity here and maintain zero net transmembrane pressure throughout, so that ion–membrane charge interactions can be examined independently of the pressure-driven water transport that we characterized separately for the same membrane models in earlier work [41]. This is particularly relevant when preferentially rejecting divalent ions over monovalent ones is crucial. To accomplish this, we employ methods for simulating charged NF membranes that we recently developed [8,42]. This allows us to compare single ion feed solutions (LiCl or MgCl_2_) to mixed feed solutions (LiCl and MgCl_2_) to understand how pH-induced membrane charge affects ion transport with particular emphasis on improving Li^+^/Mg^2+^ selectivity. Throughout, we use the terms ion transport behavior and ion penetration behavior interchangeably to describe the molecular-scale movement of ions within the membrane, which contribute to macroscopic selectivity. By elucidating the molecular-level mechanisms underlying Li^+^/Mg^2+^ selectivity, this study provides mechanistic insight to guide the rational design of NF membranes optimized for lithium separation. More broadly, the results demonstrate how pH-dependent functional group chemistry and ion competition jointly control mono/divalent ion selectivity in charged polymeric membranes, advancing the development of next-generation functional materials for selective ion separations.

## 2. Materials and Methods

Details of membrane construction and the MD simulations are provided elsewhere [8,42], so we only provide an overview here. The first step is to build a charge-neutral membrane model starting with 332 piperazine (PIP) and 228 trimesoyl chloride (TMC) monomers that are randomly packed in a 52 × 52 × 50 Å^3^ simulation reaction box, resulting in a virtually polymerized membrane nanostructure of similar size with periodic boundaries in the spanwise *x*- and *y*-directions. A 52 Å cross-section is used during polymerization to ensure adequate monomer packing and crosslinking, but after equilibration, the membrane is rescaled to a 50 × 50 Å^2^ cross-section for the production simulations, matching the dimensions of the feed and permeate reservoirs. Next the neutral membrane model is charged via amine group protonation and carboxyl group deprotonation, where the number densities and distributions of NH_2_^+^ and ${\mathrm{COO}}^{-}$ functional groups at pH = 2, 7, and 10 are based on experimental measurements of the total number densities and ionization fractions of the functional groups at each pH using a silver-binding methodology [23,24]. The spatial arrangement of the charged groups within the membrane nanostructure is then assigned during the virtual polymerization by distributing groups randomly at those measured densities, as we have previously described in detail [42]. The membrane models are equilibrated using counter-ions to neutralize the charged functional groups within the membrane nanostructure. The average density of the membrane models is 0.80 ± 0.03 g ${\mathrm{cm}}^{-3}$ across the densest regions with a mean pore diameter of 5.6 ± 0.3 Å, based on PoreBlazer v4.0 [43]. These values fall within the range reported experimentally for loose polyamide NF membranes, where active layer densities below 1 g cm^−3^ and mean pore diameters of roughly 4–8 Å are typical [41]. A detailed characterization of the membrane models including net charge, charged group composition, density, and porosity are provided in our previous publication [8].

Although several assumptions are necessary to construct these active layer membrane models, they provide a range of membrane charge distributions that allows the detailed exploration of how various membrane charge conditions influence the interactions of membrane charge with ions in the feed solutions, which is the focus of this study. Several limitations of the model are worth noting. The virtual polymerization procedure produces a statistically representative nanostructure but does not capture the density gradients or local charge heterogeneities of real interfacially polymerized membranes. The charge distributions are fixed at each pH based on experimental measurements rather than dynamically fluctuating, and the system cross-section (∼50 × 50 Å^2^) is small relative to real membrane active layers. These simplifications are appropriate for isolating pH-dependent charge effects on competing ions, but absolute transport rates and sensitivity to local structural variability should be interpreted accordingly.

**Figure 1:**
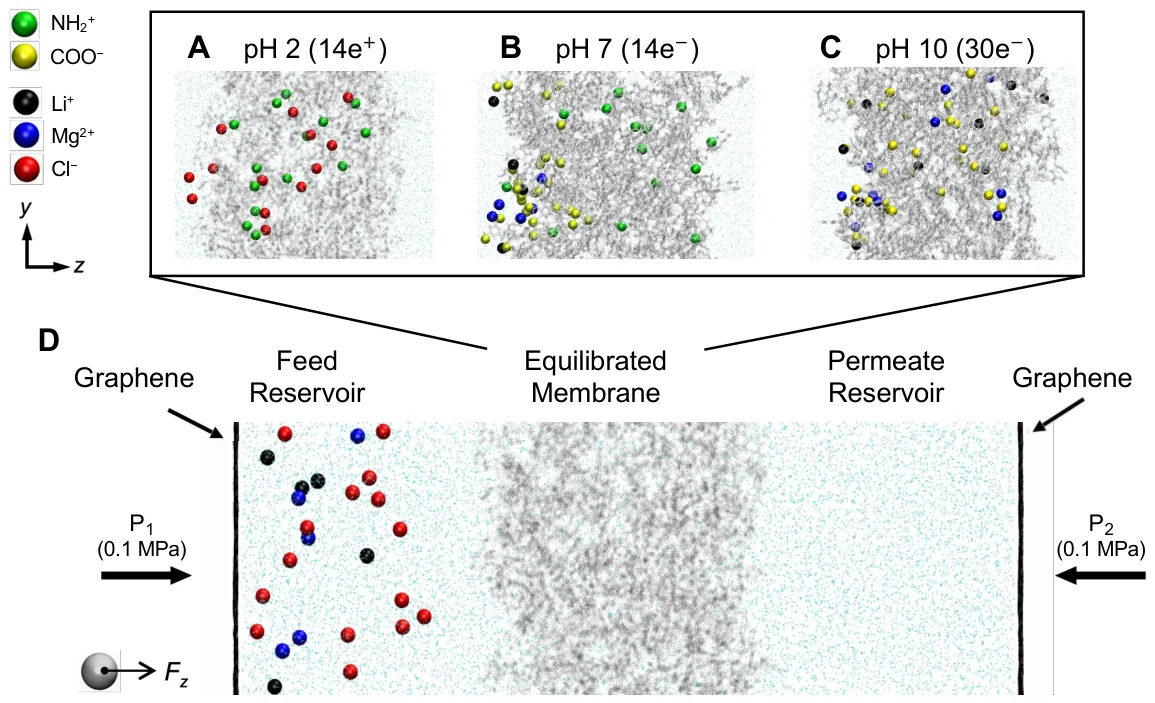
The equilibrated and hydrated molecular dynamics membrane models at (**A**) pH 2 (net charge of 14e^+^); (**B**) pH 7 (net charge of 14e^−^); and (**C**) pH 10 (net charge of 30e^−^). Note that the functional groups are distributed in the *x*-direction through the membrane, even though they may appear to be in the same *y*-*z* plane in the figure. Counter-ions are added to yield electrostatically neutral membrane systems followed by equilibration, where 14 ${\mathrm{Cl}}^{-}$ ions (red beads) are added to (**A**) pH 2 membrane system; 4 Li^+^ ions (black beads) along with 5 Mg^2+^ ions (blue beads) are added to (**B**) pH 7 membrane system; and 10 Li^+^ ions (black beads) along with 10 Mg^2+^ ions (blue beads) are added to (**C**) pH 10 membrane system. The equilibrated membrane system is used in solute feed transport simulation setup (**D**), which includes a feed reservoir (shown here is a mixture of LiCl and MgCl_2_ consisting of 5 Li^+^, 5 Mg^2+^, and 15 ${\mathrm{Cl}}^{-}$), a permeate reservoir (pure water), and two graphene sheets on the outer boundary of either reservoir to render zero transmembrane pressure. Here, water molecules are represented by light blue dots, graphene sheets are colored black and the membrane is represented by the light gray matrix.

After equilibration of the hydrated charged membrane nanostructure, the membrane model is placed between identical feed and permeate reservoirs (50 × 50 × 45 Å^3^, after further equilibration) and matching the dimensions of the membrane in the *x*- and *y*-directions with periodic boundary conditions, as depicted in Figure 1. In addition to the water molecules already present in the membrane during hydration and equilibration, the feed and permeate reservoirs are filled with water molecules packed at approximately 1 g cm^−3^. Single-layer graphene sheets bounding each reservoir bear an externally applied pressure of 0.1 MPa, thus maintaining a net zero transmembrane pressure. Ions are initially in the feed reservoir. The number of ions for the dilute solutions for each of three simulations are as follows: for LiCl there are 10 Li^+^ and 10 ${\mathrm{Cl}}^{-}$ ions; for MgCl_2_ there are 10 Mg^2+^ and 20 ${\mathrm{Cl}}^{-}$ ions; for the mixed feed there are 5 Li^+^, 5 Mg^2+^, and 15 Cl^−^ ions. The concentrated solutions have five times as many ions (for instance the mixed feed has 25 Li^+^, 25 Mg^2+^, and 75 Cl^−^ ions). The permeate reservoir contains only water molecules initially. The counter-ions added during the equilibration process are initially in vicinity of membrane charges; however, they can move freely during simulations. After a typical 100 ns simulation, most counter-ions remain within 5 Å of the membrane charged groups.

Since solute feed ions cannot penetrate the membrane within a reasonable amount of simulation time (75 ns at a transmembrane pressure of 600 MPa), we facilitate solute ion transport by applying a body force in the positive *z*-direction to each feed ion [40,44,45]. The magnitude of this force is proportional to the ion’s Coulombic charge, effectively simulating a potential difference of 0.5 V across the simulation box [42]. Specifically, a force of 0.072 kcal mol^−1^Å^−1^ is exerted on monovalent ions (Li^+^ and ${\mathrm{Cl}}^{-}$) and 0.144 kcal mol^−1^Å^−1^ on divalent ions (Mg^2+^). This approach ensures accelerated ion movement through the membrane without causing ion clustering at the membrane surface or disrupting the membrane structure [42]. Because the body force is applied proportionally according to each ion’s Coulombic charge, the relative energetic landscape experienced by Li^+^ versus Mg^2+^ is preserved; the differential electrostatic barriers that govern selectivity are therefore not expected to be artificially altered by the driving force, although quantitative transport rates are accelerated relative to typical NF membrane operating conditions. To allow the membrane to move within a small range of motion without being displaced due to force-biased solute ions, roughly 5% of the atoms in the *yz*-plane at the periodic boundaries of the membrane are fixed in space. Three independent 100 ns simulations are performed to ensure repeatability, each starting with all ions in the feed reservoir. Since we investigate solute–membrane interactions rather than water transport, which we considered previously [41], the transmembrane pressure is zero and, subsequently, the reservoir sizes remain constant during the simulation.

All simulations are conducted with the Nanoscale Molecular Dynamic (NAMD) simulation package [46], in conjunction with the General AMBER Force Field (GAFF) [47,48,49]. GAFF was chosen for consistency with our previously validated simulations of the same PIP-TMC membrane chemistry [8,42]. Its transferable parameterization for general organic systems, combined with the AM1-BCC charge scheme used for the membrane monomers, gives a consistent treatment of the polymer network alongside the separately validated ion parameters. The interactions for LiCl and MgCl_2_ are based on Lennard-Jones and Coulombic terms to capture van der Waals and electrostatic contributions [50]. The SHAKE algorithm [51] with a cutoff of the non-bonded potential of 9 Å is used to constrain the bond between each hydrogen and its mother atom. The Particle Mesh Ewald (PME) method [52] is used to compute full electrostatics with a grid spacing of 1 Å. The time step is set at 1 fs with output saved every 2 ps. The water model used here is TIP4P [53], which results in reasonable water transport performance and ion dynamics in NF membrane simulations [54]. The global temperature is set to 300 K, where the Langevin damping coefficient constant is 2 ${\mathrm{ps}}^{-1}$.

To determine which water molecules to include in the hydration shell for water coordination number measurements, the Radial Distribution Function (RDF) is calculated for all ion-water oxygen pairs [54], and the cutoff distance for water molecules in the first hydration shell is set as the radius in the RDF corresponding to the first valley after the first peak in the RDF. The cutoff distance is 2.5 Å for Li^+^, 2.4 Å for Mg^2+^, and 3.6 Å for ${\mathrm{Cl}}^{-}$, similar to other recent studies [41,55,56,57].

## 3. Results and Discussion

### 3.1. Solute Transport

**Figure 2:**
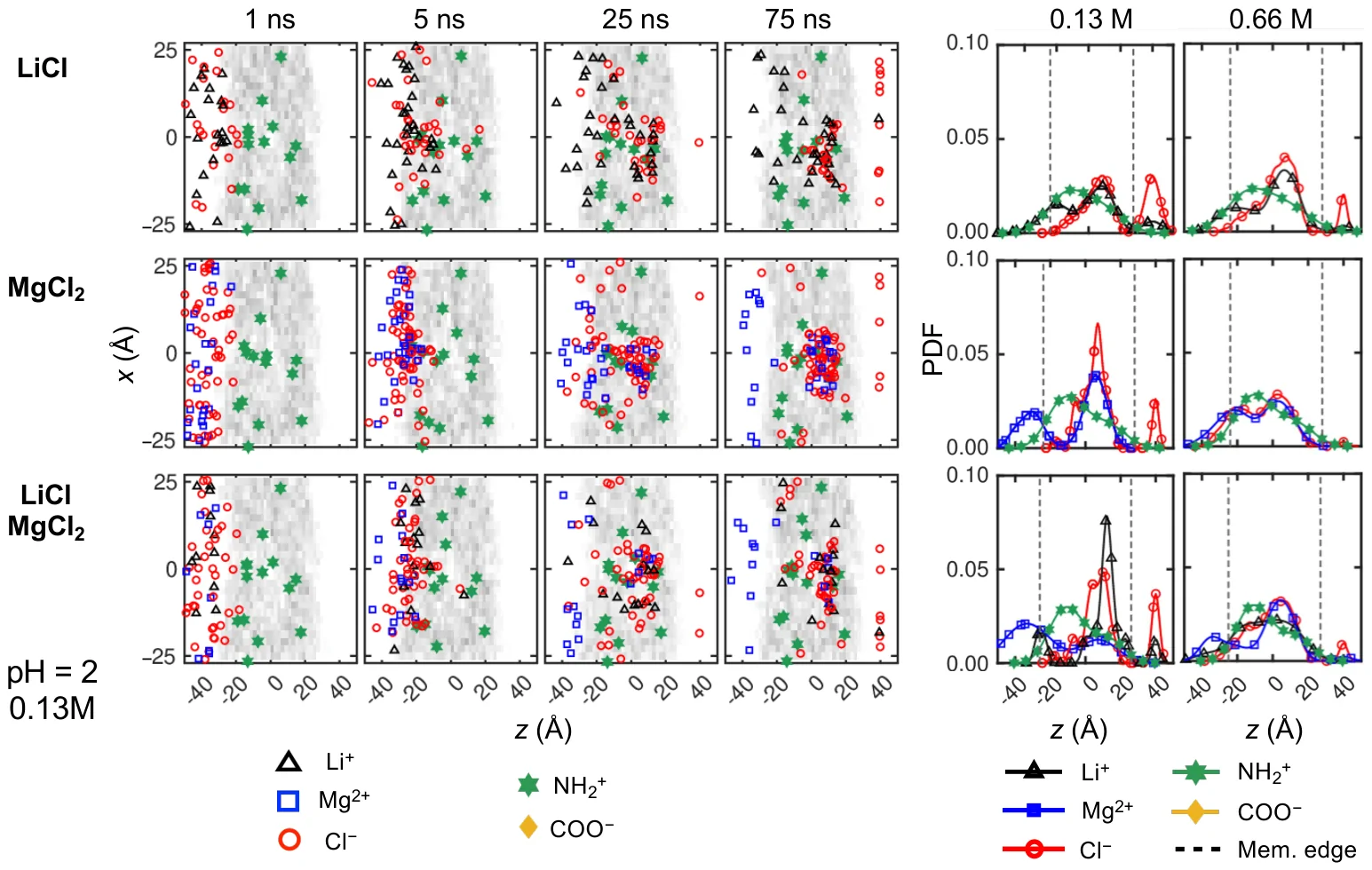
Feed ion locations for 0.13 M LiCl and 0.13 M MgCl_2_ feed solutions and a mixed 0.065 M LiCl and 0.065 M MgCl_2_ feed solution (rows) for left-to-right transport overlaid for three trials for pH = 2 at 1, 5, 25, and 75 ns (columns 1–4). Instantaneous ion and charged group locations are projected onto the *xz*-plane but are at varying depthwise *y*-locations. The *x*-axis is stretched compared to the *z*-axis to more clearly show the distribution of ions and charged end groups (ions reaching the permeate reservoir are reset to *z* = 40 Å). The membrane is centered at z=0, and darker gray indicates higher membrane density. Columns 5 and 6 show distributions (PDFs) of feed ions and membrane functional groups in the *z*-direction at 75 ns for 0.13 M and 0.66 M feed solutions based on a default kernel smoothing function in MATLAB R2025b (The MathWorks, Inc., Natick, MA, USA) (50% of default bandwidth for ${\mathrm{Cl}}^{-}$ and 80% for the cations’ default bandwidths) [58,59,60,61]. Symbols identify each curve but do not represent individual data points. Dashed vertical lines mark the membrane boundaries.

**Figure 3:**
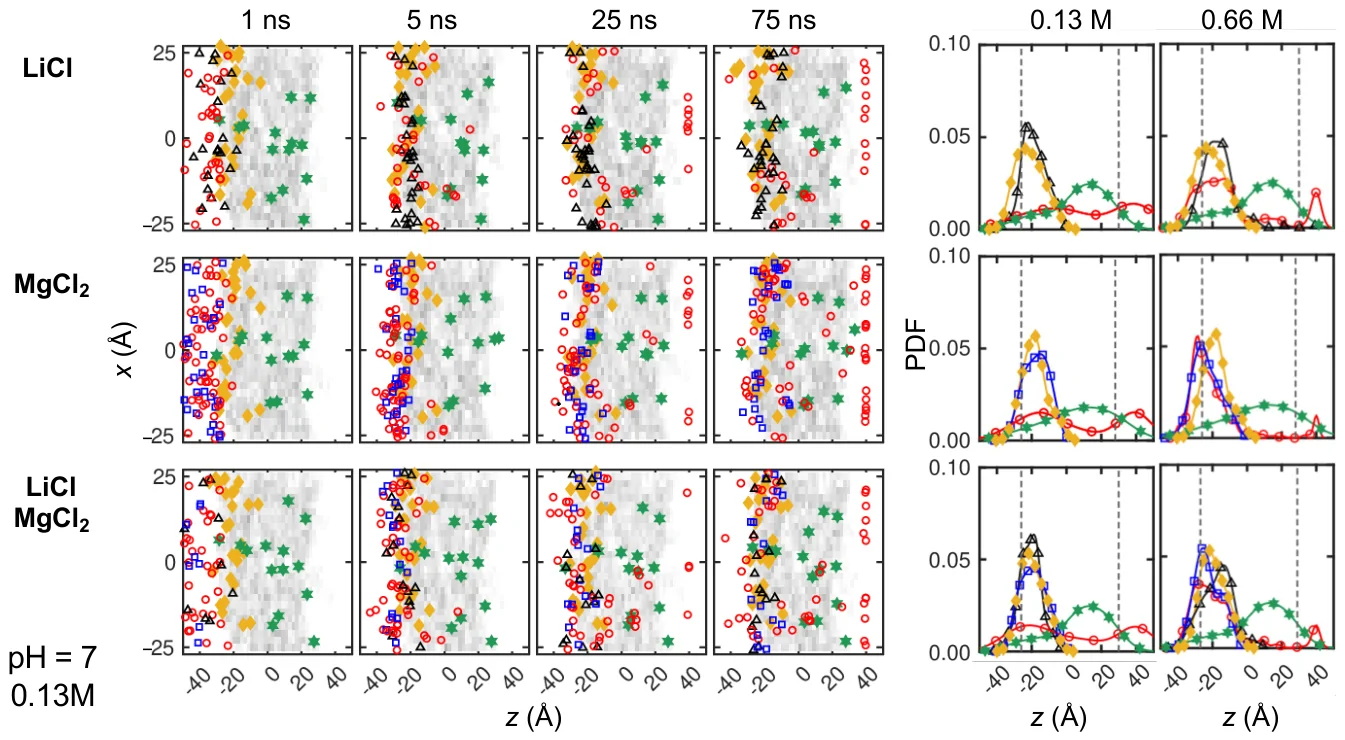
Feed ion locations for 0.13 M LiCl and 0.13 M MgCl_2_ feed solutions and a mixed 0.065 M LiCl and 0.065 M MgCl_2_ feed solution (rows) for three trials at pH = 7. Columns 5 and 6 show PDFs of ion and functional group *z*-locations. See Figure 2 caption for details.

**Figure 4:**
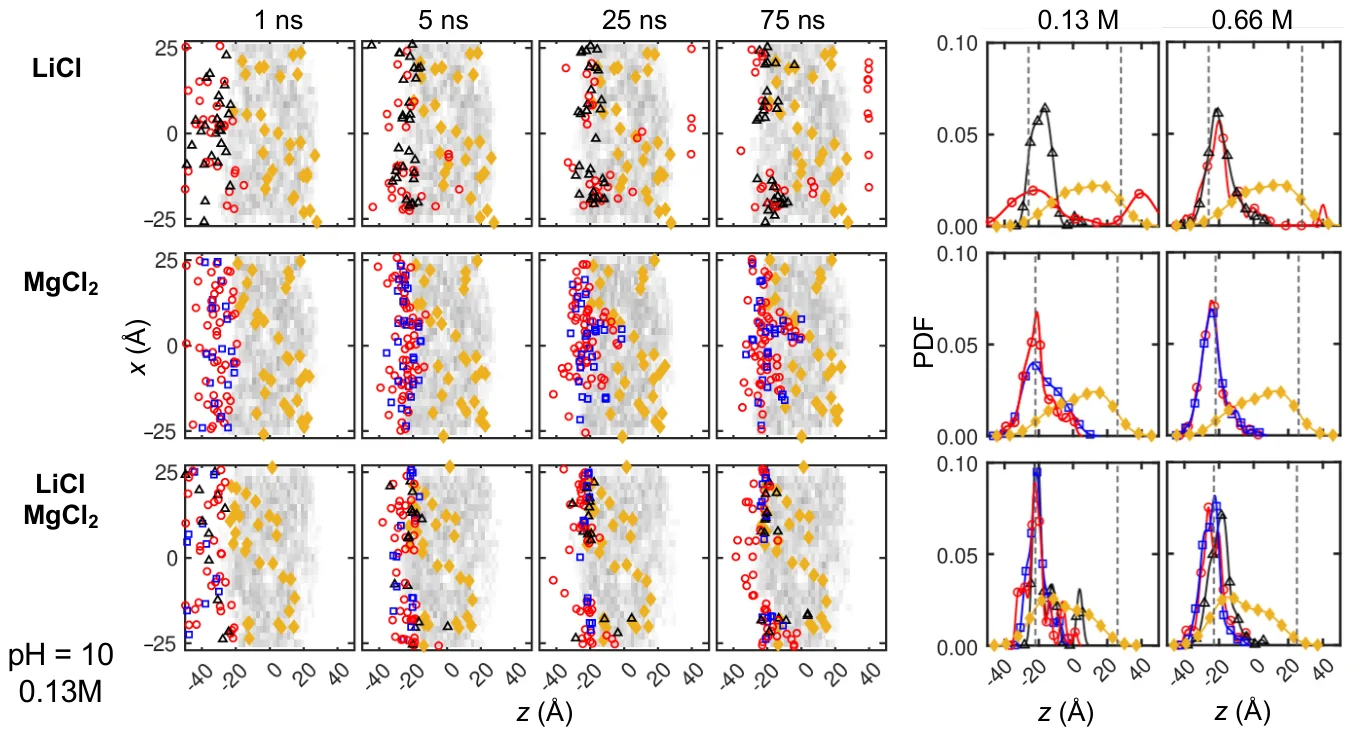
Feed ion locations for 0.13 M LiCl and 0.13 M MgCl_2_ feed solutions and a mixed 0.065 M LiCl and 0.065 M MgCl_2_ feed solution (rows) for three trials at pH = 10. Columns 5 and 6 show PDFs of ion and functional group *z*-locations. See Figure 2 caption for details.

We consider feed solutions at concentrations typical of salt lake brine [8]: 0.13 M LiCl and 0.13 M MgCl_2_ single cation feed solutions and a mixed 0.065 M LiCl and 0.065 M MgCl_2_ feed solution at pH = 2, 7 and 10. Throughout this section, Li^+^/Mg^2+^ selectivity is assessed from the relative ion penetration depths and depth probability distributions: a membrane is considered selective when the Li^+^ distribution is shifted more deeply into or through the membrane compared to the Mg^2+^ distribution under the same feed conditions. Membrane charge resulting from amine group protonation (NH_2_^+^) and carboxyl group deprotonation (${\mathrm{COO}}^{-}$) at these pH levels is based on based experimental measurements [23,24] as described previously [8,42]. Feed ion and charged group locations are projected onto the *xz*-plane at 1 ns, 5 ns, 25 ns, and 75 ns after starting in the feed reservoir across three trials in Figure 2, Figure 3, and Figure 4 for pH = 2, 7 and 10, respectively. Probability density functions (PDFs) of ions and functional groups at 75 ns are shown for the feed concentrations above and five times greater.

First, consider Li^+^ and ${\mathrm{Cl}}^{-}$ (total of 30 Li^+^ and 30 ${\mathrm{Cl}}^{-}$ ions over three simulations) transport at pH = 2 (top row, Figure 2), where NH_2_^+^ groups are distributed throughout the positively-charged membrane. Initially (*t* = 1 ns), most ions are near the feed surface, with a few outside of the frame of the image. Li^+^ ions are slightly further from the membrane due to repulsion from NH_2_^+^ groups in the membrane. By *t* = 5 ns, ${\mathrm{Cl}}^{-}$ ions progress into the membrane, attracted by the NH_2_^+^ groups, while Li^+^ ions are nearer the surface. At 25 ns, most ${\mathrm{Cl}}^{-}$ ions are in the membrane, but several Li^+^ ions remain outside of the membrane. By 75 ns, some ${\mathrm{Cl}}^{-}$ ions transit through the membrane with Li^+^ ions following behind to balance the charge. A video of the transport process suggests that some ${\mathrm{Cl}}^{-}$ ions carry Li^+^ ions along, following roughly the same path, similar to NaCl at pH = 2 [42].

For MgCl_2_ (second row, Figure 2), the initial progress of 30 Mg^2+^ and 60 ${\mathrm{Cl}}^{-}$ ions through the membrane is slower than LiCl, because the Mg^2+^ valency results in stronger NH_2_^+^ repulsion. By 25 ns, Mg^2+^ and ${\mathrm{Cl}}^{-}$ ions accumulate in a void region of the membrane (0≤z≤15 Å, −10≤x≤5 Å), where the local membrane density is only 0.2 g cm^−3^, compared to the typical density of 0.8 g cm^−3^. This void is a local feature of the membrane polymeric nanostructure rather than a typical pore as characterized above using PoreBlazer, which is based on a structural average over the whole equilibrated membrane. Results for 10 and 15 ns indicate that Cl^−^ ions first traverse to the void due to favorable Cl^−^–NH_2_^+^ interactions and then facilitate Mg^2+^ transport, eventually leading to clustering of 26 Cl^−^ ions and 12 Mg^2+^ ions in the void (Figure S1 of the Supplementary Material), noting that these counts represent the cumulative number of distinct ions across three independent simulation trials; not all of the ions are simultaneously within the void at one instant. By 75 ns, a few Cl^−^ ions reach the permeate reservoir, while Mg^2+^ ions remain in the feed reservoir or clustered with Cl^−^ ions in the void. The qualitative similarity to CaCl_2_ at pH = 2 [42] suggests that electrostatic charge effects dominate.

For a mixed LiCl-MgCl_2_ feed (third row, Figure 2, 15 Li^+^, 15 Mg^2+^, and 45 ${\mathrm{Cl}}^{-}$ ions over three simulations), attractive NH_2_^+^ interactions initially lead to fast ${\mathrm{Cl}}^{-}$ transport compared to cations (1 ns). At 5 and 25 ns, ${\mathrm{Cl}}^{-}$ ions penetrate further into the membrane, with monovalent Li^+^ ions following along. Divalent Mg^2+^ ions have difficulty overcoming NH_2_^+^ repulsion, remaining near the membrane surface, unlike the pure MgCl_2_ solution (second row, Figure 2, accounting for half as many Mg^2+^ ions in the mixed case). ${\mathrm{Cl}}^{-}$ ions can chaperone either cation species through the membrane for single cation feed solutions but tend to chaperone Li^+^ ions for the mixed feed. Apparently, strong Mg^2+^-NH_2_^+^ repulsion provides an electrostatic barrier, consistent with previous experiments [28]. By 25 ns, Li^+^ ions and a few Mg^2+^ ions accumulate in the low-density void, even though most Mg^2+^ ions have not yet entered the membrane. This clustering likely results in steric hindrance for Mg^2+^ ions, further slowing their movement through the membrane. By 75 ns, some ${\mathrm{Cl}}^{-}$ ions reach the permeate and most Li^+^ ions are deep in the membrane, while Mg^2+^ ions are either stuck in the void or still in the feed reservoir.

Clearly, transport for Li^+^ and Mg^2+^ depends on whether both ions are present or not. For single cations, both species are similarly rejected, either not entering the membrane at all or stuck in the void. However, for a mixed feed, Li^+^ ions penetrate the membrane more easily than Mg^2+^ ions, clearly evident in the spatial PDFs at 75 ns (column 5, Figure 2). Single cation feed solutions have bimodal distributions with the overlapping peaks for Li^+^ or Mg^2+^ with ${\mathrm{Cl}}^{-}$ suggesting that ${\mathrm{Cl}}^{-}$ ions facilitate cation transport past repulsive NH_2_^+^ groups, consistent with experimental evidence [28]. For the mixed feed, the Li^+^ and Mg^2+^ distributions are still bimodal, but within the membrane the Li^+^ peak is larger and the Mg^2+^ peak is smaller than the single cation cases, with the Mg^2+^ distribution skewed toward the membrane feed surface. Thus, Mg^2+^ transport is preferentially hindered at the membrane surface by the NH_2_^+^ charge, while ${\mathrm{Cl}}^{-}$ ions preferentially escort Li^+^ ions into and through the membrane, resulting in selective Li^+^ transport at the expense of Mg^2+^ transport.

Increasing the feed concentration by a factor of 5 profoundly affects Li^+^/Mg^2+^ selectivity (last column, Figure 2). For LiCl, the distributions of Li^+^ and ${\mathrm{Cl}}^{-}$ ions for 0.13 M and 0.66 M are similar. In contrast, Mg^2+^ peaks in the distribution at 0.66 M are less distinct, and ${\mathrm{Cl}}^{-}$ ions are distributed through the membrane, presumably due to electrostatic attraction to Mg^2+^ ions, which have difficulty traversing the membrane due to repulsive NH_2_^+^ interactions. The effect of concentration for the mixed feed is more profound: the Li^+^/Mg^2+^ selectivity that occurs at low feed concentration is substantially reduced, consistent with experiments [8,28]. Preferential Li^+^ transport through the membrane decreases at high concentration and more Mg^2+^ ions penetrate into the membrane. Close examination of the feed ion locations at high concentration for 5 and 25 ns (Figure S4 of the Supplementary Material) indicates that ${\mathrm{Cl}}^{-}$ ions penetrate the membrane more quickly at high concentration with both cations following them, rather than mostly Li^+^ ions following them at low concentration. The ion–membrane interaction energy landscape, which is discussed in detail in the next section, further supports this. The interaction energies are nearly identical at high and low concentrations (see the next section and Figure S7), indicating that direct electrostatic screening of membrane charge by additional counter-ions is not the primary driver of reduced selectivity. Rather, the reduced selectivity appears to stem from the less preferential ${\mathrm{Cl}}^{-}$ escorting at high concentration, where a greater number of Mg^2+^–${\mathrm{Cl}}^{-}$ clusters compete for pore entry alongside Li^+^.

When the pH increases from 2 to 7 (Figure 3), ${\mathrm{COO}}^{-}$ groups are near the feed surface with NH_2_^+^ groups deeper in the membrane, which dramatically changes ion transport. For all three feed solutions, both Li^+^ and Mg^2+^ ions remain near the feed surface, while some ${\mathrm{Cl}}^{-}$ ions permeate through the membrane within 25 ns, presumably drawn past repulsive ${\mathrm{COO}}^{-}$ groups by attractive NH_2_^+^ groups deeper in the membrane. By 75 ns, the cation and ${\mathrm{COO}}^{-}$ distributions coincide, as counter-ions associate with oppositely-charged functional groups, while co-ions easily pass the near-surface charged groups, as found previously for Na^+^ and Ca^2+^ [42]. The result is substantially reduced Li^+^/Mg^2+^ selectivity at pH = 7, consistent with experiments [8,28]. Higher feed concentrations yield similar results, although more ${\mathrm{Cl}}^{-}$ ions are trapped with cations near the feed surface due to steric effects and electrostatic interactions (Figure S5 of the Supplementary Material). Unlike pH = 2, Li^+^/Mg^2+^ selectivity at pH = 7 is poor regardless of concentration, consistent with experiments [8,28].

At pH = 10 (Figure 4), the membrane is negatively charged, having ${\mathrm{COO}}^{-}$ groups distributed throughout the membrane with no NH_2_^+^ groups at all. The simulations do not include reactive chemistry, so the possible solution-phase formation of insoluble hydroxide species such as Mg(OH)_2_ at pH = 10 is not represented. The membrane charge at this pH reflects only the deprotonation state of the polymer functional groups, and any precipitation that might occur in a real feed solution at this pH and concentration is a separate process not captured in these simulations. Similar to pH = 7, cations are stopped near the feed surface for both single and mixed cation feeds, even though ${\mathrm{COO}}^{-}$ functional groups are deeper in the membrane at pH = 10. When Mg^2+^ ions are present, stronger Mg^2+^ electrostatic interactions reduce ${\mathrm{Cl}}^{-}$ transport. For LiCl and mixed feeds, ions at the feed surface tend to be bunched near ${\mathrm{COO}}^{-}$ groups, apparently due to cation attractive interactions. This does not occur for the MgCl_2_ feed, possibly because of differences in ion solvation, as discussed shortly. A small peak in the low concentration Li^+^ distribution near z=0 suggests slight tendency toward Li^+^/Mg^2+^ selectivity for the mixed feed. Results are similar at high feed concentrations (Figure S6 of the Supplementary Material).

To better understand the tendency for Li^+^ and Mg^2+^ ions to associate with ${\mathrm{Cl}}^{-}$ ions, we measure the ensemble-averaged ion pair lifetimes, which are 3.9 ns, 0.6 ns, and 1.0 ns at pH = 2, 7, and 10 for Li^+^–${\mathrm{Cl}}^{-}$ but somewhat longer at 5.6 ns, 2.5 ns and 4.4 ns for Mg^2+^–${\mathrm{Cl}}^{-}$. This may result in clusters, particularly for Mg^2+^–${\mathrm{Cl}}^{-}$, that may be larger than the average membrane pore size (≈5.6 Å), leading to steric repulsion. While the pair lifetimes for Li^+^ ions are shorter, the attraction may lead to ${\mathrm{Cl}}^{-}$ ions chaperoning Li^+^ through the membrane. The shorter Li^+^–${\mathrm{Cl}}^{-}$ lifetime at pH = 2 (3.9 ns) enables ${\mathrm{Cl}}^{-}$ ions to escort Li^+^ forward and detach more readily, whereas the longer-lived Mg^2+^–${\mathrm{Cl}}^{-}$ pairs (5.6 ns) form complexes that tend to be sterically excluded from the pore. Of course, during the nanosecond timescale of our simulations, the chaperoning effect competes with electrostatic hindrance by ${\mathrm{COO}}^{-}$ groups. In contrast, ${\mathrm{Cl}}^{-}$ ions can traverse the membrane by hopping from one NH_2_^+^ to another at pH = 2 or pH = 7, although no NH_2_^+^ groups at pH = 10 hinders ${\mathrm{Cl}}^{-}$ transport.

### 3.2. Ion–Membrane Interaction Energy

**Figure 5:**
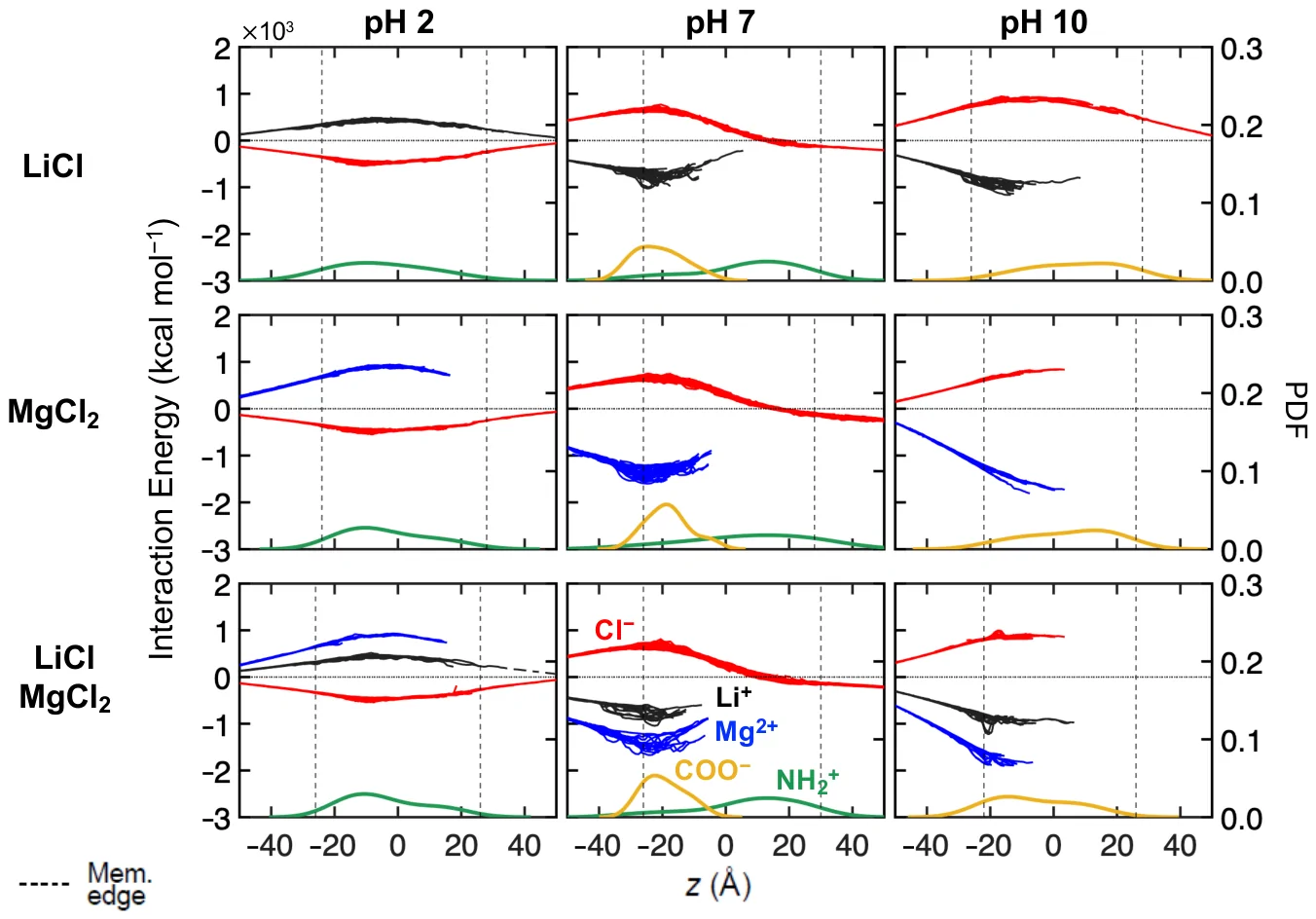
Interaction energy between feed ions and the membrane charge across the membrane thickness (*z*-direction) for all trials at the low concentration with pH = 2, 7, and 10 for LiCl (top row), MgCl_2_ (middle row), and mixed (bottom row) solutions. PDFs for NH_2_^+^ (green) and ${\mathrm{COO}}^{-}$ (yellow) groups are included in the lower portion of each panel; note that at pH = 2 only the NH_2_^+^ PDF (green) appears since there are no ${\mathrm{COO}}^{-}$ groups, and at pH = 10 only the ${\mathrm{COO}}^{-}$ PDF (yellow) appears since there are no NH_2_^+^ groups. Dashed vertical lines indicate the edge-to-edge boundaries of the membrane model. The *z*-axis extends from the feed reservoir through the membrane to the permeate reservoir, with the membrane centered at z=0. The number of overlaid curves corresponds to the number of ions at each condition, which extend only to *z*-locations that ions have sampled in the membrane.

To disentangle charge interactions from steric effects, we characterize the ion–membrane interaction energy landscape using the NAMDEnergy tool to compute non-bonding interactions (electrostatic and van der Waals) [8,42]. The computations assume a vacuum, and neither steric effects nor water solvation are included. This vacuum approximation isolates the direct ion-functional group electrostatic landscape but excludes solvation and entropic contributions, so it should not be interpreted as a total free energy of ion transfer. The hydration analysis in Section 3.3 provides the complementary solvation picture that the NAMDEnergy calculation omits. Interaction energies for all of the ions in all three trials over 100 ns simulations are shown in Figure 5 for low concentration feed solutions.

In all cases, interaction energies increase from the feed reservoir moving toward the membrane, with the sign depending on the membrane’s pH-dependent charge distribution. For example, at pH = 2 for LiCl, the interaction energy becomes increasingly positive for Li^+^ and negative for ${\mathrm{Cl}}^{-}$ due to repulsive and attractive interactions with NH_2_^+^ groups, respectively. Interaction energies are largest where NH_2_^+^ density is highest and decrease as ions move past the NH_2_^+^ groups. At pH = 7, the interaction energies have opposite sign and are largest where ${\mathrm{COO}}^{-}$ groups are concentrated. The ${\mathrm{Cl}}^{-}$ interaction energy changes sign deeper in the membrane where the attractive interactions with NH_2_^+^ groups become stronger with some ${\mathrm{Cl}}^{-}$ ions reaching the permeate reservoir. Variations in the Li^+^ interaction energy, evident as spread for the overlaid curves, results from varying distances from specific charged groups with overlapping curves for *z* > −20 Å where fewer Li^+^ ions penetrate deeply into the membrane. Interaction energies for pH = 10 are slightly larger due to the overall negative charge of the membrane, and the maxima locations reflect the location of the ${\mathrm{COO}}^{-}$ groups. The ${\mathrm{Cl}}^{-}$ interaction energies for MgCl_2_ are similar to those for LiCl but do not extend as far to the right because ions do not penetrate as far, while those for Mg^2+^ are approximately double that for Li^+^ because of their charge. The interaction energies for the mixed feed (bottom row, Figure 5) are nearly the same as those for single solute feed solutions. This is unsurprising because the interaction energy measures the interaction of individual solute ions with the overall charge of the membrane, which depends only on pH, not which solutes are present, and therefore consistent with previous measurements [8,42]. Interaction energies at higher feed concentrations (Figure S7 of the Supplementary Material) are nearly identical except that the curves do not extend as far to the right due to higher rejection.

### 3.3. Ion Solvation

**Figure 6:**
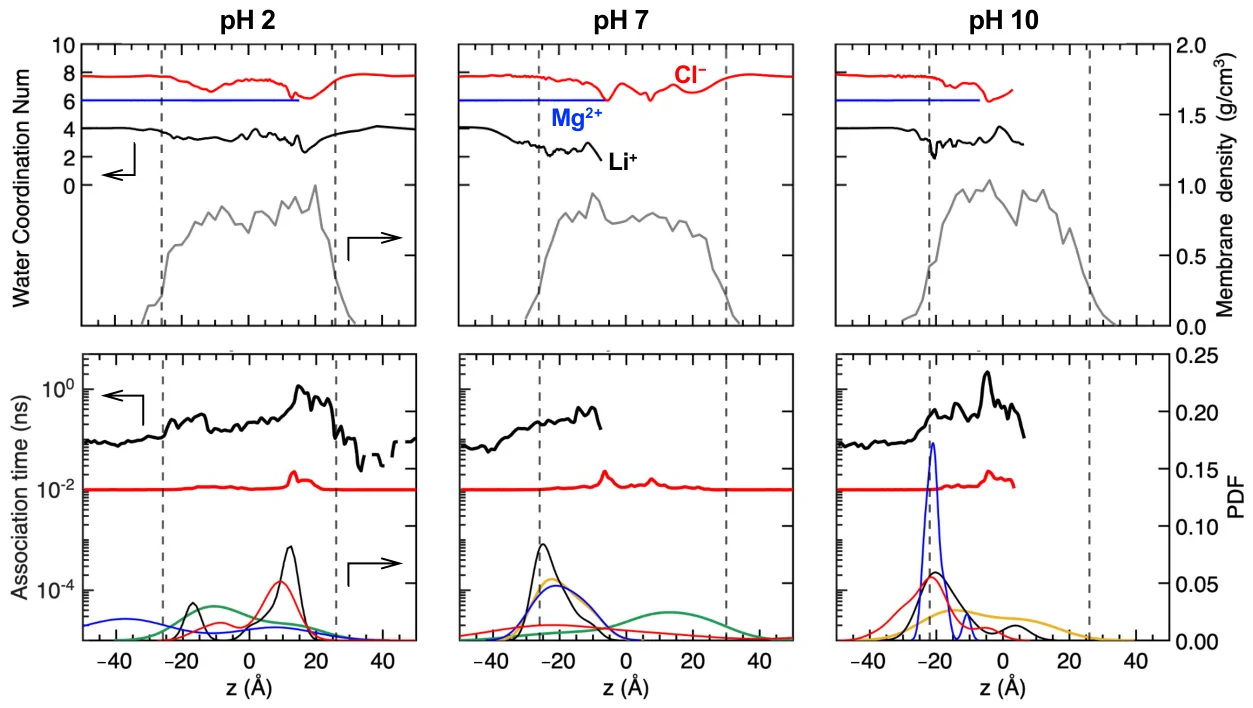
Average water coordination number and membrane density (top row) as well as water association time (bottom row) in the reservoirs and through the membrane thickness (*z*-direction) for all three trials for the low concentration mixed solution with pH = 2, 7, and 10. PDFs for NH_2_^+^ (green) and ${\mathrm{COO}}^{-}$ (yellow) groups in the membrane along with PDFs for Li^+^, Mg^2+^, and ${\mathrm{Cl}}^{-}$ ions at 75 ns are included in the lower portion of bottom row (see Figure 2 for color key). Dashed vertical lines indicate the edge-to-edge boundaries of the membrane model. The right end of each curve reflects the deepest penetration of the ions.

The hydration shell resulting from electrostatic interaction of an ion with water dipoles influences ion rejection [39,52,62] due to steric effects related to the hydrated ion size: 4.28 Å for Mg^2+^, compared to 3.32 Å for ${\mathrm{Cl}}^{-}$ and 3.82 Å for Li^+^ [63,64]. Both Li^+^ and ${\mathrm{Cl}}^{-}$ shed one or two water molecules within the membrane based on water coordination numbers (average number of water molecules within the first solvation shell), shown in the top row of Figure 6. The fewest solvation shell water molecules correspond to denser portions of the membrane. In contrast, Mg^2+^ does not shed any water molecules because of its large solvation energy (437.5 kcal/mol) [65]. This has a direct consequence for selectivity: Mg^2+^ retains its full hydration shell as ions move into the membrane, so its hydrated size (∼4.28 Å) is comparable to the mean pore diameter (5.6 Å), imposing steric resistance on top of the electrostatic repulsion from NH_2_^+^ groups. Li^+^, by contrast, sheds one or two water molecules in the membrane, reducing its effective size and allowing it to pass through pore constrictions that may exclude the more strongly solvated Mg^2+^, thereby amplifying the selectivity advantage that electrostatic interactions alone would produce.

The average association time for water molecules in the solvation shell is an order of magnitude longer for Li^+^ ions than ${\mathrm{Cl}}^{-}$ ions and further increases where membrane density is largest and coordination number is smallest, as shown in the bottom row of Figure 6. Longer association times reflect reduced water exchange dynamics associated with strong ion hydration, as previously reported for divalent cations such as Mg^2+^ [66]. Under nanoconfinement, restricted water mobility may further slow hydration shell reorganization. Mg^2+^ ions do not shed water molecules within the simulation time because of their much higher solvation energy than Li^+^ (113.5 kcal/mol) or ${\mathrm{Cl}}^{-}$ ions (81.2 kcal/mol) [65].

## 4. Conclusions

Ion selectivity in polyamide membranes depends the combination of pH-dependent charge, the presence of other ions, and feed concentration. For an acidic feed (pH = 2), positive membrane charge assists ${\mathrm{Cl}}^{-}$ transport, which draws cations along despite of NH_2_^+^ repulsion. However, when both Li^+^ and Mg^2+^ are present at low concentrations, Mg^2+^ ions are more strongly repelled by NH_2_^+^ groups than Li^+^ ions leading to reduced Mg^2+^ transport and better Li^+^/Mg^2+^ selectivity; at high concentrations, this effect is significantly reduced. At pH =7, ${\mathrm{COO}}^{-}$ groups near the membrane feed surface prevent cations from traversing further into the membrane, although ${\mathrm{Cl}}^{-}$ ions are drawn by NH_2_^+^ groups deeper in the membrane. When the feed is basic (pH = 10), cations are stopped at the negatively-charged membrane surface by attractive interactions with ${\mathrm{COO}}^{-}$ groups and ${\mathrm{Cl}}^{-}$ ions remain co-located with these cations near the feed surface, consistent with the similar ion penetration depths evident in the interaction energy profiles in Figure 5, since there are no attractive NH_2_^+^ groups present at this pH to draw ${\mathrm{Cl}}^{-}$ further into the membrane. In both cases, Li^+^/Mg^2+^ selectivity is substantially reduced by the negative membrane charge even if it is present only at the feed surface. Thus, Li^+^/Mg^2+^ selectivity is optimal at low feed concentrations and under positive membrane charge, at least for the short transient of ∼100 ns possible using MD simulations.

While the macroscopic role of membrane charge in ion selectivity has been studied experimentally, what happens at the molecular scale during ion-functional group interactions is inaccessible to experiment. This is precisely the gap that MD simulations fill. Among the specific contributions of this work are a molecular-level characterization of membrane charge interactions across a range of pH conditions, including at pH = 10 where strongly negative membrane charge essentially arrests ion transport, and a demonstration that mixed-ion competitive effects, particularly the differential repulsion of Mg^2+^ versus Li^+^ by NH_2_^+^ groups in a mixed feed, cannot be inferred from single-salt simulations alone. Based on how charged ${\mathrm{COO}}^{-}$ and NH_2_^+^ groups in the membrane interact with Li^+^, Mg^2+^, and ${\mathrm{Cl}}^{-}$ ions, positively charged membranes—whether due to pH-dependent membrane protonation or from intrinsic membrane charge unrelated to pH—are optimal for lithium concentration applications, at least for the conditions that were simulated here (feed concentrations of 0.065–0.13 M, PIP-TMC polyamide membranes at the charge states studied, and with the constraint of the ∼100 ns timescale accessible to MD). Of course, the selectivity may also depend on ion concentrations, membrane chemistry, and operating conditions. Furthermore, these simulations are performed at zero net transmembrane pressure specifically to isolate ion–membrane charge interactions from water transport, which was already established in our earlier work [41]. Because this selectivity behavior is governed by charge-driven ion-functional group interactions rather than by pressure-driven flow, the resulting mechanistic trends should transfer directly to practical membrane design regardless of the operating pressure used.

More broadly, these findings demonstrate how charged functional groups govern mixed-ion separation selectivity in polymeric membranes beyond lithium recovery. The mechanistic insights presented here are broadly applicable to charge-selective separations such as water treatment, desalination, and resource recovery from complex ionic mixtures, underscoring the importance of molecular-level understanding in the design of next-generation functional membrane materials.

## Data Availability

The data presented in this study are available on request from the corresponding author.

## Supplementary Materials

The following supporting information can be downloaded at https://www.mdpi.com/article/10.3390/membranes16080264/s1: Figure S1: Feed ion locations for LiCl, MgCl_2_, and mixed LiCl/MgCl_2_ solutions at intermediate times at pH = 2; Figure S2: Feed ion locations for LiCl, MgCl_2_, and mixed LiCl/MgCl_2_ solutions at intermediate times at pH = 7; Figure S3: Feed ion locations for LiCl, MgCl_2_, and mixed LiCl/MgCl_2_ solutions at intermediate times at pH = 10; Figure S4: Feed ion locations for LiCl, MgCl_2_, and mixed LiCl/MgCl_2_ solutions at high concentration at pH = 2; Figure S5: Feed ion locations for LiCl, MgCl_2_, and mixed LiCl/MgCl_2_ solutions at high concentration at pH = 7; Figure S6: Feed ion locations for LiCl, MgCl_2_, and mixed LiCl/MgCl_2_ solutions at high concentration at pH = 10; Figure S7: Interaction energy between feed ions and membrane charges at high concentration for pH = 2, 7, and 10.

## References

[1] Li J., Peng H., Liu K., Zhao Q. (2023). Polyester nanofiltration membranes for efficient cations separation. Adv. Mater..

[2] Sarkar P., Modak S., Karan S. (2021). Ultraselective and highly permeable polyamide nanofilms for ionic and molecular nanofiltration. Adv. Funct. Mater..

[3] Zheng H., Mou Z., Lim Y.J., Srikanth N., Zhang W., Guo S., Wang R., Zhou K. (2022). High-precision and high-flux separation by rationally designing the nanochannels and surface nanostructure of polyamide nanofiltration membranes. Small Sci..

[4] Ji Y.L., Gu B.X., Huo H.Q., Xie S.J., Peng H., Zhang W.H., Yin M.J., Xiong B., Lu H., Villalobos L.F. (2024). Roll-to-roll fabrication of large-area metal-organic framework-based membranes for high-performance aqueous separations. Nat. Water.

[5] Peng H., Su Y., Liu X., Li J., Zhao Q. (2023). Designing gemini-electrolytes for scalable Mg^2+^/Li^+^ separation membranes and modules. Adv. Funct. Mater..

[6] Guo S., Wan Y., Chen X., Luo J. (2021). Loose nanofiltration membrane custom-tailored for resource recovery. Chem. Eng. J..

[7] Peng H., Yu K., Liu X., Li J., Hu X., Zhao Q. (2023). Quaternization-spiro design of chlorine-resistant and high-permeance lithium separation membranes. Nat. Commun..

[8] Foo Z.H., Liu S., Kanias L., Lee T.R., Heath S.M., Tomi Y., Miyabe T., Keten S., Lueptow R.M., Lienhard J.H. (2024). Positively-coated nanofiltration membranes for lithium recovery from battery leachates and salt-lakes: Ion transport fundamentals and module performance. Adv. Funct. Mater..

[9] Das S., Deng E., Tandel A.M., Singh S., Mowatt J.V., Lin H. (2026). Nanofiltration membranes for Li^+^/Mg^2+^ separation: Materials and mechanisms. J. Polym. Sci..

[10] Wang Y., Zhang Y., Zhang M., Jiang S., Tang X., Liang H. (2025). Advanced biomimetic nanofiltration membranes for lithium recovery with anti-janus charge structure. Nat. Commun..

[11] Kiso Y., Nishimura Y., Kitao T., Nishimura K. (2000). Rejection properties of non-phenylic pesticides with nanofiltration membranes. J. Membr. Sci..

[12] Schäfer A., Nghiem L., Waite T. (2003). Removal of the natural hormone estrone from aqueous solutions using nanofiltration and reverse osmosis. Environ. Sci. Technol..

[13] Kiso Y., Sugiura Y., Kitao T., Nishimura K. (2001). Effects of hydrophobicity and molecular size on rejection of aromatic pesticides with nanofiltration membranes. J. Membr. Sci..

[14] Charcosset C. (2009). A review of membrane processes and renewable energies for desalination. Desalination.

[15] Pontié M., Dach H., Leparc J., Hafsi M., Lhassani A. (2008). Novel approach combining physico-chemical characterizations and mass transfer modelling of nanofiltration and low pressure reverse osmosis membranes for brackish water desalination intensification. Desalination.

[16] Schaep J., Van der Bruggen B., Vandecasteele C., Wilms D. (1998). Influence of ion size and charge in nanofiltration. Sep. Purif. Technol..

[17] Van der Bruggen B., Vandecasteele C., Van Gestel T., Doyen W., Leysen R. (2003). A review of pressure-driven membrane processes in wastewater treatment and drinking water production. Environ. Prog..

[18] Ng L.Y., Mohammad A.W., Ng C.Y., Leo C.P., Rohani R. (2014). Development of nanofiltration membrane with high salt selectivity and performance stability using polyelectrolyte multilayers. Desalination.

[19] Labban O., Liu C., Chong T.H., Lienhard J.H. (2018). Relating transport modeling to nanofiltration membrane fabrication: Navigating the permeability-selectivity trade-off in desalination pretreatment. J. Membr. Sci..

[20] Le N.L., Nunes S.P. (2016). Materials and membrane technologies for water and energy sustainability. Sustain. Mater. Technol..

[21] Pontié M., Derauw J., Plantier S., Edouard L., Bailly L. (2013). Seawater desalination: Nanofiltration—A substitute for reverse osmosis?. Desalin. Water Treat..

[22] Abdel-Fatah M.A. (2018). Nanofiltration systems and applications in wastewater treatment. Ain Shams Eng. J..

[23] Coronell O., González M.I., Mariñas B.J., Cahill D.G. (2010). Ionization behavior, stoichiometry of association, and accessibility of functional groups in the active layers of reverse osmosis and nanofiltration membranes. Environ. Sci. Technol..

[24] Ritt C.L., Werber J.R., Wang M., Yang Z., Zhao Y., Kulik H.J., Elimelech M. (2020). Ionization behavior of nanoporous polyamide membranes. Proc. Natl. Acad. Sci. USA.

[25] Meihong L., Sanchuan Y., Yong Z., Congjie G. (2008). Study on the thin-film composite nanofiltration membrane for the removal of sulfate from concentrated salt aqueous: Preparation and performance. J. Membr. Sci..

[26] Zhao D., Yu S. (2015). A review of recent advance in fouling mitigation of NF/RO membranes in water treatment: Pretreatment, membrane modification, and chemical cleaning. Desalin. Water Treat..

[27] Xu R., Qin W., Zhang B., Wang X., Li T., Zhang Y., Wen X. (2020). Nanofiltration in pilot scale for wastewater reclamation: Long-term performance and membrane biofouling characteristics. Chem. Eng. J..

[28] Foo Z.H., Rehman D., Bouma A.T., Monsalvo S., Lienhard J.H. (2023). Lithium concentration from salt-lake brine by Donnan-enhanced nanofiltration. Environ. Sci. Technol..

[29] Kamcev J., Freeman B.D. (2016). Charged polymer membranes for environmental/energy applications. Annu. Rev. Chem. Biomol. Eng..

[30] Rivas B.L., Pereira E.D., Palencia M., Sánchez J. (2011). Water-soluble functional polymers in conjunction with membranes to remove pollutant ions from aqueous solutions. Prog. Polym. Sci..

[31] Wang X.L., Shang W.J., Wang D.X., Wu L., Tu C.H. (2009). Characterization and applications of nanofiltration membranes: State of the art. Desalination.

[32] Mohammad A.W., Teow Y., Ang W., Chung Y., Oatley-Radcliffe D., Hilal N. (2015). Nanofiltration membranes review: Recent advances and future prospects. Desalination.

[33] Bowen W.R., Jenner F. (1995). Theoretical descriptions of membrane filtration of colloids and fine particles: An assessment and review. Adv. Colloid Interface Sci..

[34] Mason E., Lonsdale H. (1990). Statistical-mechanical theory of membrane transport. J. Membr. Sci..

[35] Ghidossi R., Veyret D., Moulin P. (2006). Computational fluid dynamics applied to membranes: State of the art and opportunities. Chem. Eng. Process. Process Intensif..

[36] Ridgway H.F., Orbell J., Gray S. (2017). Molecular simulations of polyamide membrane materials used in desalination and water reuse applications: Recent developments and future prospects. J. Membr. Sci..

[37] Heiranian M., DuChanois R.M., Ritt C.L., Violet C., Elimelech M. (2022). Molecular simulations to elucidate transport phenomena in polymeric membranes. Environ. Sci. Technol..

[38] Shen M., Keten S., Lueptow R.M. (2016). Dynamics of water and solute transport in polymeric reverse osmosis membranes via molecular dynamics simulations. J. Membr. Sci..

[39] Shen M., Keten S., Lueptow R.M. (2016). Rejection mechanisms for contaminants in polyamide reverse osmosis membranes. J. Membr. Sci..

[40] Luo Y., Harder E., Faibish R.S., Roux B. (2011). Computer simulations of water flux and salt permeability of the reverse osmosis FT-30 aromatic polyamide membrane. J. Membr. Sci..

[41] Liu S., Ganti-Agrawal S., Keten S., Lueptow R.M. (2022). Molecular insights into charged nanofiltration membranes: Structure, water transport, and water diffusion. J. Membr. Sci..

[42] Liu S., Foo Z.H., Lienhard J.H., Keten S., Lueptow R.M. (2025). Membrane charge effects on solute transport nanofiltration: Experiments and molecular dynamics simulations. Membranes.

[43] Sarkisov L., Bueno-Perez R., Sutharson M., Fairen-Jimenez D. (2020). Materials informatics with PoreBlazer v4.0 and the CSD MOF database. Chem. Mater..

[44] Ding M., Szymczyk A., Ghoufi A. (2015). On the structure and rejection of ions by a polyamide membrane in pressure-driven molecular dynamics simulations. Desalination.

[45] Leung K., Rempe S.B. (2009). Ion rejection by nanoporous membranes in pressure-driven molecular dynamics simulations. J. Comput. Theor. Nanosci..

[46] Phillips J.C., Braun R., Wang W., Gumbart J., Tajkhorshid E., Villa E., Chipot C., Skeel R.D., Kale L., Schulten K. (2005). Scalable molecular dynamics with NAMD. J. Comput. Chem..

[47] Wang J., Wolf R.M., Caldwell J.W., Kollman P.A., Case D.A. (2004). Development and testing of a general AMBER force field. J. Comput. Chem..

[48] Jakalian A., Jack D.B., Bayly C.I. (2002). Fast, efficient generation of high-quality atomic charges. AM1-BCC model: II. Parameterization and validation. J. Comput. Chem..

[49] Wang J., Wang W., Kollman P.A., Case D.A. (2006). Automatic atom type and bond type perception in molecular mechanical calculations. J. Mol. Graph. Model..

[50] Beglov D., Roux B. (1994). Finite representation of an infinite bulk system: Solvent boundary potential for computer simulations. J. Chem. Phys..

[51] Ryckaert J.P., Ciccotti G., Berendsen H.J. (1977). Numerical integration of the Cartesian equations of motion of a system with constraints: Molecular Dynamics of n-alkanes. J. Comput. Phys..

[52] Darden T., York D., Pedersen L. (1993). Particle Mesh Ewald: An Nlog(N) method for Ewald sums in large systems. J. Chem. Phys..

[53] Jorgensen W.L., Chandrasekhar J., Madura J.D., Impey R.W., Klein M.L. (1983). Comparison of simple potential functions for simulating liquid water. J. Chem. Phys..

[54] Liu S., Keten S., Lueptow R.M. (2023). Effect of molecular dynamics water models on flux, diffusivity, and ion dynamics for polyamide membrane simulations. J. Membr. Sci..

[55] Belch A.C., Berkowitz M., McCammon J. (1986). Solvation structure of a sodium chloride ion pair in water. J. Am. Chem. Soc..

[56] Bruni F., Imberti S., Mancinelli R., Ricci M. (2012). Aqueous solutions of divalent chlorides: Ions hydration shell and water structure. J. Chem. Phys..

[57] Yamaguchi T., Ohzono H., Yamagami M., Yamanaka K., Yoshida K., Wakita H. (2010). Ion hydration in aqueous solutions of lithium chloride, nickel chloride, and caesium chloride in ambient to supercritical water. J. Mol. Liq..

[58] Bowman A.W., Azzalini A. (1997). Applied Smoothing Techniques for Data Analysis: The Kernel Approach with S-Plus Illustrations.

[59] Peter D. (1985). Kernel estimation of a distribution function. Commun. Stat.-Theory Methods.

[60] Jones M.C. (1993). Simple boundary correction for kernel density estimation. Stat. Comput..

[61] Silverman B.W. (2018). Density Estimation for Statistics and Data Analysis.

[62] Yoon Y., Lueptow R.M. (2005). Removal of organic contaminants by RO and NF membranes. J. Membr. Sci..

[63] Nightingale E.R. (1959). Phenomenological theory of ion solvation. Effective radii of hydrated ions. J. Phys. Chem..

[64] Zhong C., Deng Y., Hu W., Qiao J., Zhang L., Zhang J. (2015). A review of electrolyte materials and compositions for electrochemical supercapacitors. Chem. Soc. Rev..

[65] Zhang H., Hou J., Hu Y., Wang P., Ou R., Jiang L., Liu J.Z., Freeman B.D., Hill A.J., Wang H. (2018). Ultrafast selective transport of alkali metal ions in metal organic frameworks with subnanometer pores. Sci. Adv..

[66] Schwierz N. (2020). Kinetic pathways of water exchange in the first hydration shell of magnesium. J. Chem. Phys..

