# Supplementary material for: Charged Functional Groups Drive Nanofiltration Li+/Mg2+ Selectivity
Source: Membranes (Basel). 2026 Aug 10;16(8):264. doi: 10.3390/membranes16080264 (PMC13515870; doi:10.3390/membranes16080264)
Supplement: Supplementary file 1 [file membranes-16-00264-s001.zip › membranes-4412428-supplementary.pdf]

Supplementary Materials: Charged Functional Groups Drive Nanofiltration Li^{+}/Mg^{2+} Selectivity

Suwei Liu [1], Jiaxuan (Jenny) Wang [2], Sinan Keten [1,3] and Richard M. Lueptow [1,4,*]

1. Ion Locations at Intermediate Times

The results in Figures S1, S2, and S3 are similar to those in Figures 2–4 in the main text except that intermediate times of 10 and 15 ns are included.

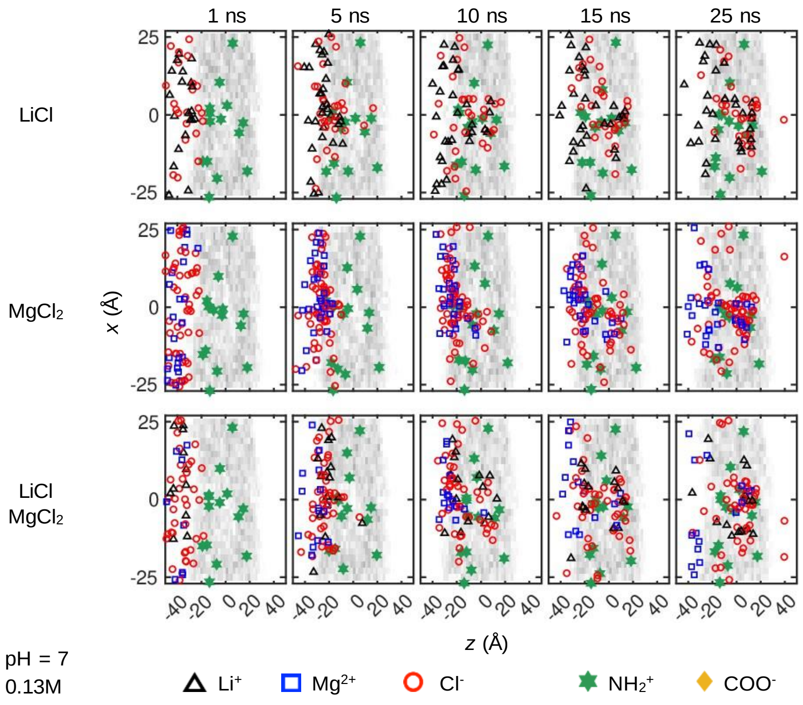

Figure S1. Feed ion locations for 0.13 M LiCl and 0.13 M $MgCl_2$ feed solutions and a mixed 0.065 M LiCl and 0.065 M $MgCl_2$ feed solution (rows) for three trials at pH = 2. See the Figure 2 caption in the main text for details.

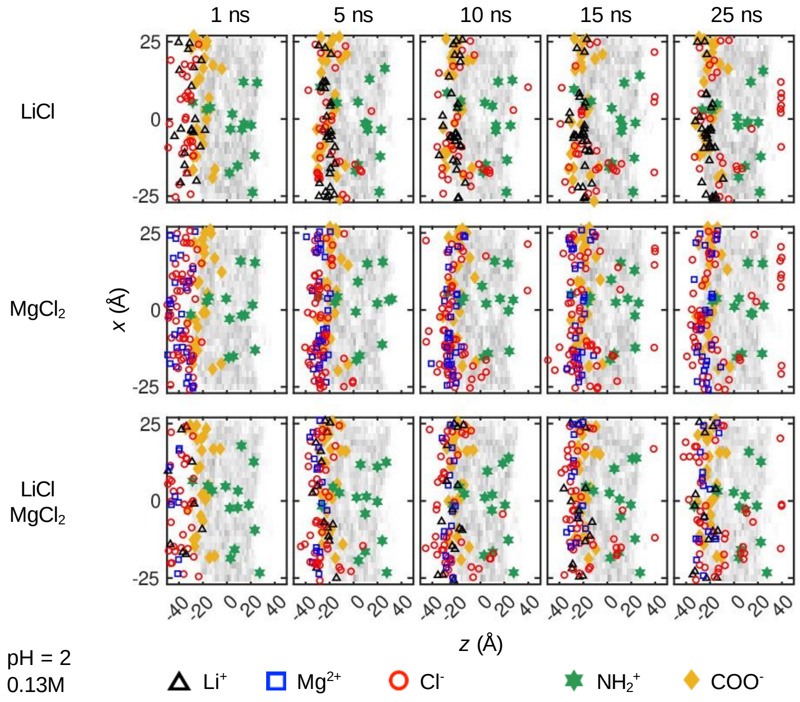

Figure S2. Feed ion locations for 0.13 M LiCl and 0.13 M $MgCl_2$ feed solutions and a mixed 0.065 M LiCl and 0.065 M $MgCl_2$ feed solution (rows) for three trials at pH = 7. See the Figure 2 caption in the main text for details.

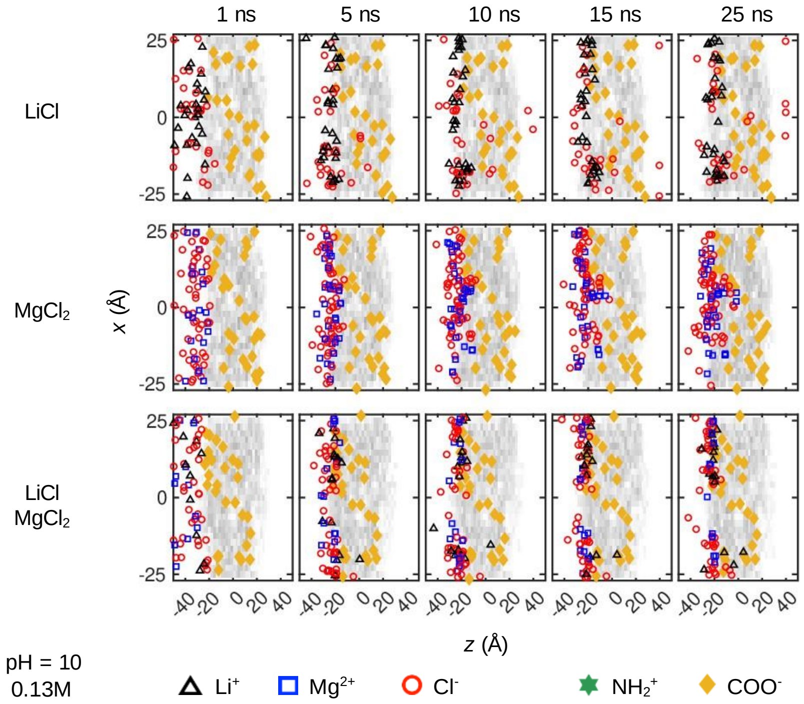

Figure S3. Feed ion locations for 0.13 M LiCl and 0.13 M $MgCl_2$ feed solutions and a mixed 0.065 M LiCl and 0.065 M $MgCl_2$ feed solution (rows) for three trials at pH = 10. See the Figure 2 caption in the main text for details.

2. High Concentration Results

The results in Figures S4, S5, and S6 are similar to those of Figures 2–4 in the main text except at feed concentrations that are 5 times higher.

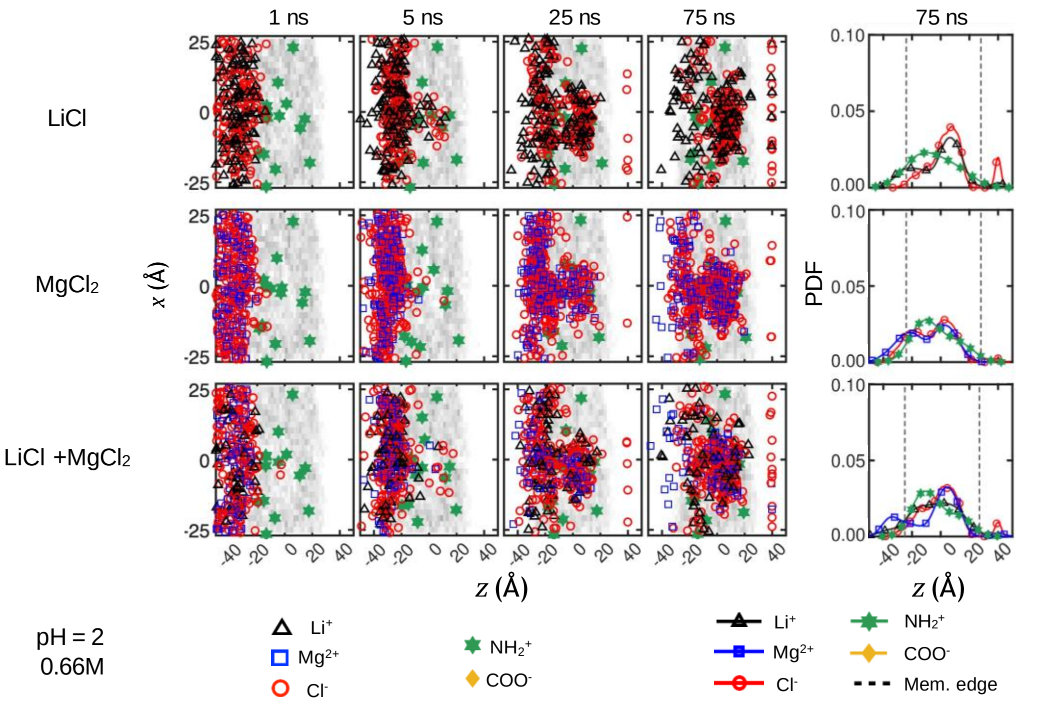

Figure S4. Feed ion locations for 0.66 M LiCl and 0.66 M $MgCl_2$ feed solutions (for each of three simulations: 50 Li^+ and 50 Cl^- for LiCl; 50 Mg^{2+} and 100 Cl^- for $MgCl_2$) and a mixed 0.33 M LiCl and 0.33 M $MgCl_2$ feed solution (for each of three simulations: 25 Li^+, 25 Mg^{2+}, and 75 Cl^-) in the xz-plane for left-to-right transport across three trials for pH = 2 at 1 ns, 5 ns, 25 ns, and 75 ns. Column 5 shows the probability density functions (PDFs) of feed ions and membrane functional groups (COO^-, NH_2^+) in the z direction at 75 ns. See the Figure 2 caption in the main text for other details.

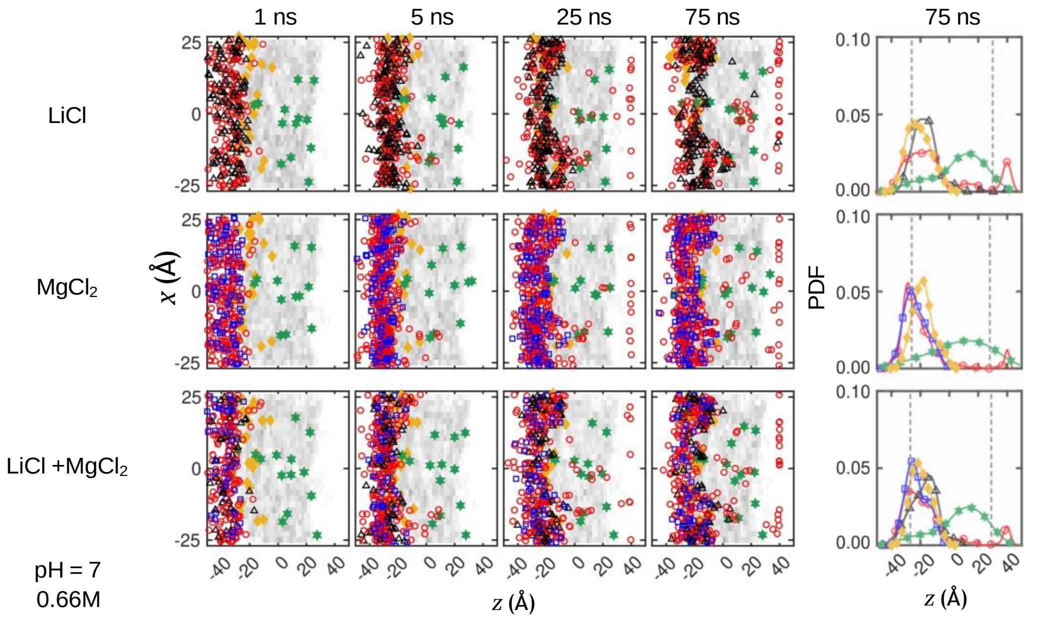

Figure S5. Feed ion locations for 0.66 M LiCl and 0.66 M $MgCl_2$ feed solutions (for each of three simulations: 50 Li^+ and 50 Cl^- for LiCl; 50 Mg^{2+} and 100 Cl^- for $MgCl_2$) and a mixed 0.33 M LiCl and 0.33 M $MgCl_2$ feed solution (for each of three simulations: 25 Li^+, 25 Mg^{2+}, and 75 Cl^-) in the xz-plane for left-to-right transport across three trials for pH = 7 at 1 ns, 5 ns, 25 ns, and 75 ns. Column 5 shows the probability density functions (PDFs) of feed ions and membrane functional groups (COO^-, NH_2^+) in the z direction at 75 ns. See the Figure 2 caption in the main text for other details.

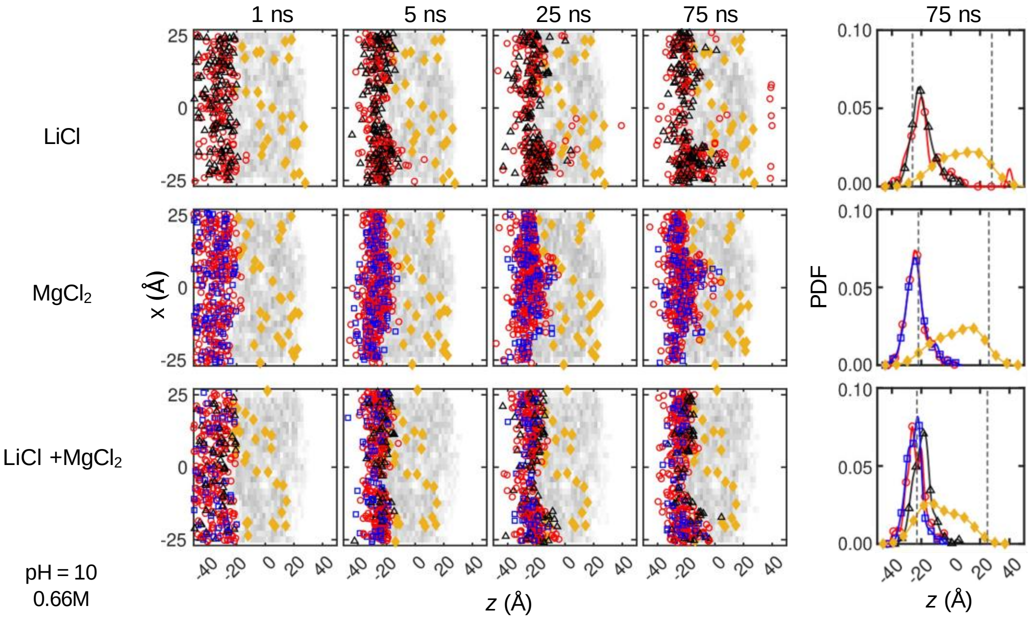

Figure S6. Feed ion locations for 0.66 M LiCl and 0.66 M $MgCl_2$ feed solutions (for each of three simulations: 50 Li^+ and 50 Cl^- for LiCl; 50 Mg^{2+} and 100 Cl^- for $MgCl_2$) and a mixed 0.33 M LiCl and 0.33 M $MgCl_2$ feed solution (for each of three simulations: 25 Li^+, 25 Mg^{2+}, and 75 Cl^-) in the *xz*-plane for left-to-right transport across three trials for pH = 10 at 1 ns, 5 ns, 25 ns, and 75 ns. Column 5 shows the probability density functions (PDFs) of feed ions and membrane functional groups (COO^-, NH_2^+) in the *z* direction at 75 ns. See the Figure 2 caption in the main text for other details.

3. Ion-Membrane Interaction Energy at High Concentration

The results in Figure S7 for the ion-membrane interaction energy at high concentration are similar to those in Figure 5 in the main text at low concentration.

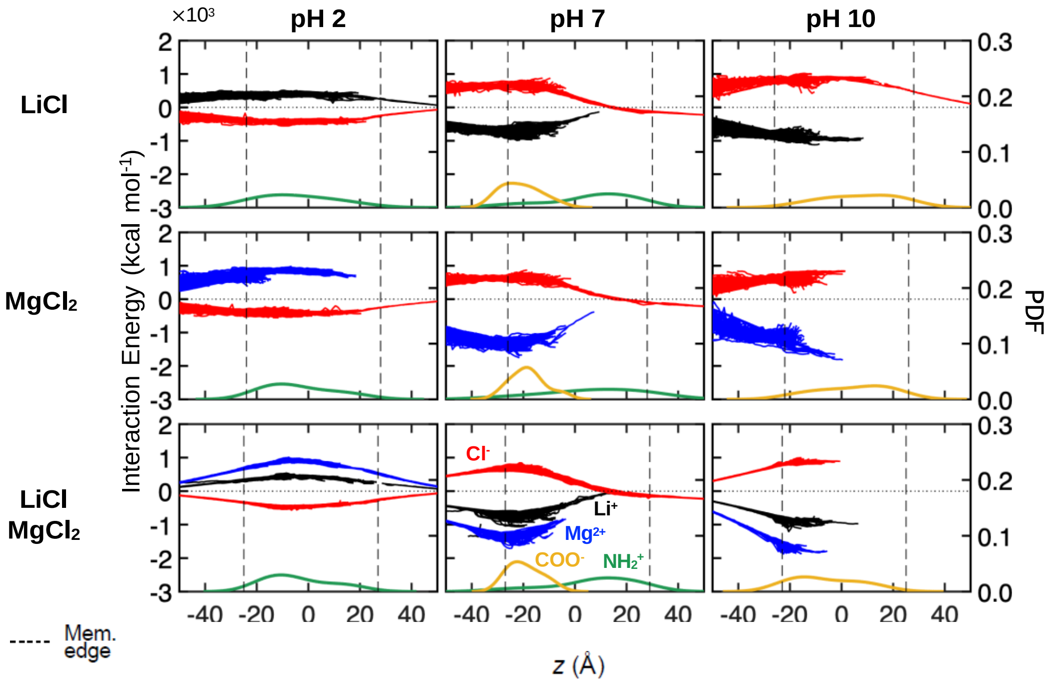

Figure S7. Interaction energy between feed ions and the membrane charge across the membrane thickness (z-direction) for all trials at the high feed concentration with pH = 2, 7, and 10 for LiCl (top row), $MgCl_2$ (middle row), and mixed (bottom row) solutions. PDFs for NH_2^+ (green) and COO^- (yellow) groups are included in the lower portion of each panel. Dashed vertical lines indicate the edge-to-edge boundaries of the membrane model. The number of overlaid curves corresponds to the number of ions at each condition, which extend only to z-locations that ions have sampled in the membrane.
